# K-D Balance: An objective measure of balance in tandem and double leg stances

**DOI:** 10.1177/2055207619885573

**Published:** 2019-11-04

**Authors:** Chelsea Zhang, Alexandra Talaber, Melanie Truong, Bert B Vargas

**Affiliations:** 1 Department of Neurology, University of Texas Southwestern Medical Center, Dallas, USA; 2 King-Devick Technologies, Inc., Oakbrook Terrace, USA

**Keywords:** Balance assessment, mobile device, application, balance deficit, neurological condition

## Abstract

**Background and objective:**

Subjective grade-based scoring balance assessments tend to be lengthy and have demonstrated poor repeatability and reliability. This study examined the reliability of a mobile balance assessment tool and differences in balance measurements between individuals at risk for a balance deficit secondary to a diagnosed neurological or musculoskeletal condition and a control group of healthy individuals.

**Methods:**

Objective balance testing was measured using K-D Balance on a compatible iPhone. Seventy-seven participants were enrolled (control group, *n *=* *44; group at risk for balance deficits, *n *=* *33). Mean and standard deviation of K-D Balance were recorded for each stance. Intra-rater reliability was calculated by repeating the trial.

**Results:**

Overall balance scores were superior for the control group compared with the group at risk for balance deficits in double leg stance (mean (SD): 0.15 (0.12) versus 0.18 (0.13), *p *=* *0.260), tandem stance right leg (mean (SD): 0.27 (0.17) versus 0.45 (0.49), *p *=* *0.028), and tandem stance left leg (mean (SD): 0.26 (0.17) versus 0.35 (0.35), *p *=* *0.136). Intra-rater reliability was good to excellent for K-D Balance double leg stance (intra-class correlation coefficient (ICC) = 0.80, 95% confidence interval (CI) 0.58–1.03), tandem stance right leg (ICC = 0.96, 95% CI 0.86–1.06) and tandem stance left leg (ICC = 0.98, 95% CI 0.95–1.0).

**Conclusions:**

K-D Balance revealed differences in balance performance between healthy individuals compared with individuals with neurological or musculoskeletal impairment. Objective balance measures may improve the accuracy and reliability of clinical balance assessment by detecting subtle differences in balance and aid in early detection of diseases that impair balance.

## Introduction

Balance assessments are a valuable clinical tool for monitoring neurological and musculoskeletal status as well as for managing fall risk. Balance disorders occur in up to 60% of individuals following a traumatic brain injury^1^ and in up to 50% in the general geriatric population.^2^ Strokes may lead to serious balance impairment as a result of hemiplegia or hemiparesis.^3^ Poor balance is a major risk factor for falling and tends to worsen with aging. Neurological conditions like multiple sclerosis, Parkinson’s disease, Alzheimer’s disease, and dementia can progressively impair postural stability. An estimated 60% of those with Parkinson’s disease experience a fall.^4^ Individuals with Alzheimer’s disease have an increased risk of falls, and studies have found that motor changes that impair balance may precede cognitive symptoms of the disease.^5^^,^^6^ Falls are a critical health concern as one in five falls lead to serious injury.^7^ Not only are individuals experiencing a reduction in quality of life and functioning due to a sequela of falls, but the medical costs associated with the falls are an estimated $30 billion in the United States alone.^8^ Motor control and balance issues are frequently overlooked or explained by signs of aging; however, subtle balance abnormalities could be early manifestations of disease which can be difficult to detect with current clinical tools.

Effective and easy to implement balance assessments would benefit frontline healthcare personnel who care for individuals that are at risk for falls and those with neurological conditions. At-home monitoring of balance would also be helpful to the individual who is prone to experiencing poor stability or who is at increased fall risk. Traditionally, balance is measured with subjective-scoring methods including the Balance Error Scoring System (BESS), the Romberg Test, the unipedal stance test (UST), the Berg Balance Scale (BBS), and the Performance-Oriented Mobility Assessment (POMA). The Romberg test is recommended specifically for the assessment of static balance which is impaired in the various ataxic syndromes, and the UST is also limited since it has been shown to vary in administration procedures resulting in reduced discrimination.^9^ The Timed Up and Go (TUG) test assesses functional mobility and can be used to predict fall risk.^10^ A meta-analysis that examined a large population’s TUG results concluded that the test was more reliable for determining fall risk in less healthy, lower-functioning individuals versus healthy, higher-functioning individuals.^11^ While the TUG assesses aspects of dynamic balance, it is considered a functional mobility test^10^ rather than a true measure of static balance. The BBS is one of the more well-known balance tests and has been studied in older populations and individuals with a history of stroke.^9^ The BBS is a lengthy test of 20 min that has demonstrated redundancy. Kornetti et al. showed that only 4 of the 14 items within the battery were important for reaching the cutoff point for fall risk.^12^ The POMA is another well-studied balance assessment that consists of 16 items that measure balance and gait. The POMA has been shown to have low sensitivity to change and limited responsiveness; a five-point change indicates a significant change.^10^ Similarly, the BBS also demonstrated low sensitivity to change.^13^ The BESS, BBS and POMA have a high ceiling effect, which means higher-functioning individuals frequently reach the maximum score and the results may not reflect their true balance performance.^10^

In order to improve the sensitivity of balance testing, wearable sensors, accelerometers, and gyroscopes with integrated software technology have been developed.^14^ Wearable sensors have been shown to detect differences in reduced cadence during walking, increased turn time, and increased turn-to-site time during the TUG compared with assessments taken without the sensors.^15^ A recent prospective, cross-sectional study measured gait and balance for 384 inpatients in a neurological ward and determined that wearable sensors at the ankles and lower back were clinically feasible.^16^ Additionally researchers found that 11% of the population with neurological conditions had a balance deficit.^16^

The King-Devick (K-D) Balance (King-Devick Technologies, Downers Grove, IL) was developed in order to improve the sensitivity and ease of clinical balance testing. This mobile balance assessment tool is a Food and Drug Administration–cleared balance assessment software application that provides an objective measurement of balance performance. The mobile balance assessment software is compatible with multiple generations of iPhone and iPod devices and is secured with a hands-free static device holder. The mobile balance assessment tool utilizes tri-axial coordinate data from the internal accelerometers of the mobile device to calculate a balance score. Testing consists of three stances: the double leg stance, tandem stance right foot forward, and tandem stance left foot forward. The mobile balance assessment tool does not include single leg stances in the testing procedure due to considerable variability in single leg stance measures. A large population of healthy individuals achieved a maximum number of errors on this stance even in the absence of injury.^17^ The balance assessment utilizes stances that have been used within other balance test protocols including the double leg and tandem stances within the BBS. The double leg only assesses a two feet stance. While the BBS incorporates other balance and gait aspects, the tandem stance is the most difficult item in the BBS. Passing the tandem stance within the BBS is highly associated with achieving a greater overall score, highlighting the sensitivity of the stance.^12^

The mobile balance assessment tool has been studied in sports and clinical settings. Eighty-two football players underwent testing with the mobile balance assessment tool and BESS, and comparison analysis showed that the mobile balance assessment detected errors that were undetected by BESS.^17^ The mobile balance assessment tool results from 70 healthy individuals, aged 22–61 years, found high correlation with another objective balance assessment that runs as a mobile application as well.^18^ This study’s aim was to examine the test–retest reliability of the mobile balance assessment tool and balance performance in a population of individuals with neurological or musculoskeletal disorders compared with healthy controls across a wide adult age range.

## Methods

### Participants

Participants (*n *=* *77, 24 males, 53 females), aged 18–65 years, were recruited from the University of Texas Southwestern Medical Center’s Department of Neurology. Participants were recruited to two groups: a control group of healthy individuals with no reported balance deficits (*n *=* *44, mean age 36.4 ± 11.5 years, range 20–65) and a group of individuals at a high risk for balance deficits (*n *=* *33, mean age 41.7 ± 13.9 years, range 18–65) as a result of diagnosed neurologic conditions (*n *=* *28) or musculoskeletal impairments (*n *=* *5). All participants provided written informed consent to participate in the study prior to enrollment. Study procedures were approved by the University of Texas Southwestern Institutional Review Board.

### Procedure

Participants underwent balance testing with the mobile balance assessment application on an iPhone 4. The iPhone was secured to the participant’s chest with a hands-free static device holder. The mobile balance assessment tool instructs the examiner step-by-step through the test protocol to maintain consistency with each test administration. Balance measures were completed as the participant performed three stances in the specific procedure: double leg stance (feet together), tandem stance right (standing heel-to-toe with the right foot forward), and tandem stance left (standing heel-to-toe with the left foot forward). Participants were asked to maintain each stance for 20 s with eyes closed and hands on their hips. To examine test–retest reliability, a second trial following the same procedure was completed 5 min after the initial balance assessment. K-D Balance displays numeric scores for each stance following completion of the three-stance balance assessment protocol. Three separate scores were recorded for each stance and trial. The mobile balance assessment tool displays a balance performance score for each stance. Any score above zero is indicative of movement during testing, therefore higher scores are indicative of worse balance performance.

### Statistical analysis

Statistical analyses were performed using Stata 14.2 software (StataCorp, College Station, TX). The mobile balance assessment score mean, standard deviation, and range were determined for each stance along with the difference between trials one and two. Differences in the mobile balance assessment scores between groups were compared using paired *t*-tests. Intra-class correlation coefficients (ICCs) and 95% confidence intervals (CIs) were calculated to assess the agreement between trials for the three stances. Statistical significance was set at *p *<* *0.05.

## Results

Seventy-seven participants were enrolled in the study. Participant group demographics are provided in Table 1. Mean scores by group are displayed in Table 2. The balance scores were higher (worse) for the group at risk for balance deficits compared with the control group for double leg stance (mean (SD): 0.18 (0.13) versus 0.15 (0.12), *p *=* *0.260). Similarly, tandem stance right leg balance performance was significantly worse for the group at risk for balance deficits compared with the control group (mean (SD): 0.45 (0.49) versus 0.27 (0.17), *p *=* *0.028). The balance scores were also poorer for the group at risk for balance deficits versus control group in the tandem stance left leg (mean (SD): 0.35 (0.35) versus 0.26 (0.17), *p *=* *0.136).

**Table 1.** Participant demographics by group.

	Healthy controls (*n *=* *44)	At risk for balance deficits (*n *=* *33)
Gender, % female	73%	64%
Age, mean (SD)	36.4 (11.5)	41.7 (13.9)
Age, median (range)	32.5 (20–65)	41.0 (18–65)
Diagnosis	NA	Neurological condition or brain injury (*n* = 28)Musculoskeletal injury (*n* = 5)

**Table 2.** Balance scores by group.

	Mean (SD), 95% CI	
Stance	Healthy controls (*n *=* *44)	At risk for balance deficits (*n *=* *33)
Double leg stance	0.15 (0.12)0.03–0.53	0.18 (0.13)0.04–0.49
Tandem stance right	0.27 (0.17)0.07–0.67	0.45 (0.49)0.06–2.09
Tandem stance left	0.26 (0.17)0.03–0.79	0.35 (0.35)0.08–1.61

The double leg stance had good intra-rater reliability between the mobile balance assessment trials one and two (ICC = 0.80, 95% CI 0.58–1.03). K-D Balance tandem stance right leg trials showed excellent agreement (ICC = 0.96, 95% CI 0.86–1.06). Similarly, tandem stance left leg trials had excellent agreement (ICC = 0.98, 95% CI 0.95–1.0).

## Discussion

The participants included a group of healthy individuals and a group of individuals at an elevated risk of balance deficits due to diagnosed neurological conditions or musculoskeletal injury. There were more female participants than male participants and a higher number of individuals within the age range of 23–44 years compared with individuals 45 years and over. Overall, mean balance scores were superior for the control group and worse for the group at risk for balance deficits, indicating that greater instability and corrective movements were accurately detected by the mobile balance assessment tool. Greatest differences in scores between groups were observed for the tandem stances; the group at risk for balance deficits scored worse than the control group. The double leg stance is considered to be the easiest stance out of the three-stance sequence, which explains why the scores between groups were overall better and closer together. Tandem stances are more challenging to hold compared with the double leg stance, particularly for individuals with a balance deficit, and therefore explains why there was greater disparity in scores between groups. Identifying the stances that become more challenging with balance dysfunction is important for clinicians to be aware of when screening for balance deficits that could be early manifestations of neurological or musculoskeletal disease.

The mobile balance assessment tool demonstrated excellent intra-rater reliability for tandem right and left leg stances and good intra-rater reliability for double leg stance. Instability of the hands or slight shifts in location of handheld devices can translate into motion detected by the accelerometers independent of balance instability. This is of particular importance when assessing balance in individuals with Parkinson’s disease, tremors, motor abnormalities, or movement disorders that may affect upper body strength and stability. These issues may be effectively addressed by the mobile balance assessment’s stabilizing device holder which secures the mobile device to the patient’s midline during testing and which may have contributed to the high reliability. There were individuals in the at-risk group who were diagnosed with Parkinson’s disease, myasthenia gravis, dystonia, myoclonus, and general weakness. Completion of testing on this population provides reasonable evidence that the mobile balance assessment can be performed on individuals with neurological impairment.

Other studies have demonstrated reduced balance performance in individuals with increasing age and lower physical fitness levels. A systematic review of 17 studies investigating balance performance of a healthy community-dwelling population over the age of 70 years showed that there was a significant decline in balance performance for every one-year increase in age. There was also a strong association between age and balance variability.^19^ In healthy, older individuals, risk factors for balance dysfunction include reduced physical activity, forward head posture, and increased age.^20^ Adequate musculoskeletal health is necessary to maintain normal balance as reduced balance has been shown to be a risk factor for musculoskeletal injury and poorer balance scores are indicative of lower extremity injury.^21^^,^^22^ Neurological conditions including multiple sclerosis,^23^^,^^24^ Parkinson’s disease,^25^^,^^26^ Alzheimer’s disease,^27^^,^^28^ stroke,^29^^,^^30^ essential tremor,^31^ vertigo,^32^ and traumatic brain injury and concussion^33^^,^^34^ have been shown to cause significant balance impairments compared with controls. A prospective study examined 210 community-dwelling older adults with a mean age of 80 years. Testing included the BBS, and monthly logs were completed, tracking any falls over a one-year period. It was found that not all items of the BBS were sensitive to identifying fall risk. One leg and tandem stances identified the largest number of participants as having deficits, with 88% having an impairment in one leg stance and tandem stances.^35^ Our findings similarly showed that there was greater impairment or worse tandem stance scores in the group at risk for balance deficits. The combination of aging and prevalence of these neurological conditions explains why balance dysfunction is quite common in the geriatric population and highlights the value of assessing balance for managing changes in health status and for monitoring fall risk.

Physical therapists commonly use the single leg stance and BBS to assess posture, stability, and functional balance.^36^ In a systematic review of the BBS, a 14-point balance assessment, researchers found that the test had acceptable reliability but warned that it might not identify clinically significant changes in individuals. Additionally, the analysis showed a substantial ceiling effect for participants within the cohort (*n *=* *668).^37^ Similar to the mobile balance assessment tool, the BBS has demonstrated high test–retest reliability.^38^ However, in another systematic review, authors recommended that clinicians consider using the BBS with other balance measures due to floor and ceiling effects.^38^ Balance measures that are more objective may be more sensitive in identifying subtle abnormalities or changes from baseline and help reduce the floor and ceiling effect. A major disadvantage of the BBS is that it can take up to 30 min, which is not always possible in the examination room. In a comparison study between BBS and the Static Balance Test, researchers found that the Static Balance Test was more reliable and took less time compared with the BBS, and both tests were in statistical agreement.^39^ Kim and Kim examined the reliability of the short-form BBS (a seven-item abbreviated version) in an institutionalized, geriatric population and found an intra-rater reliability of 0.83 and inter-rater reliability of 0.79,^40^ which was lower than the intra-rater reliability of the mobile balance assessment results in this study.

Newer balance tests have been developed and studied, such as balance testing with a Wii Balance Board (WBB), inertial sensors, and the TekScan MatScan®. Studies examining test–retest reliability of the WBB for individuals following a stroke demonstrate high test–retest reliability (ICC = 0.82–0.98); however, there are poor correlations between WBB portions and clinical tests.^41^ The research on the WBB is still ongoing and needs further development for validation. Numerous studies have investigated balance testing with inertial sensors which, similar to the mobile balance assessment tool, provide objective measures based on linear acceleration and gyroscopic recordings of angular velocity during balance testing. Howcroft et al. reviewed 40 studies that used inertial sensors, typically incorporating data from a gyroscope and/or accelerometer, for the evaluation of geriatric fall risk. There was lack of analysis and reporting of reliability measures in this review.^42^ Additionally, there was a wide range of sensitivity and specificity results due to the variation in populations and models used for analysis. The authors concluded that further research is needed to support these findings toward identifying a set of inertial sensor–based variables that “yield a robust and accurate fall risk assessment model and clinical tool.”^42^ The TekScan MatScan® records center of pressure in antero-posterior and medio-lateral direction directly from floor mat sensors. TekScan MatScan® has been shown to have fair to good reliability in adults (ICC = 0.44–0.95).^43^ Reliability of testing in a small population of individuals (mean age was 69 years) with rheumatoid arthritis ranged from 0.84 to 0.92.^44^ There is a lack of research on this balance test in the geriatric populations and individuals with neurological conditions; it is difficult to compare the reliability of TekScan MatScan® due to the lack of studies in various subpopulations.

### Future perspective

The authors note some limitations to this study. Leg dominance plays an important role in balance performance^45^ and the participant’s dominant leg was not recorded potentially impacting comparisons between right and left leg tandem stances. Future studies that include the recording of leg dominance would provide further information particularly in the presence of asymmetric ability between the right and left sides. Other study limitations were the lack of participants over the age of 65 years and the fact that participants were not determined in advance of the study to have subjective balance complaints or diagnosis of a balance impairment. Lastly, these data were obtained from a limited patient sample from one clinic; future studies should include a larger sample size with age-matched controls, and greater representation of the general population.

Despite a number of balance tests that are available, future studies are essential to determine the most accurate and reliable clinical assessment. A fundamental next step in this area of balance research is to examine how specific conditions impair balance. Objective balance assessments appear to detect subtle abnormalities and have the potential to aid in determining early stages of diseases that impair balance. Comparisons should also be analyzed between the mobile balance assessment tool and current clinical assessments of fall risk to determine whether or not the inclusion of this new balance assessment would improve sensitivity, specificity, and abnormal balance detection.

## Conclusion

The mobile balance assessment tool demonstrated high test–retest reliability and scores differentiated healthy controls from individuals at risk of balance deficits secondary to neurological or musculoskeletal disorders. Future studies should assess how objective balance measures compare with current clinical tests and explore the relationship between these balance scores and fall risk. Objective and reliable balance assessments using the mobile balance assessment tool have the potential to enhance the detection of subtle balance deficits, allowing for diagnostic evidence witnessed in certain medical conditions, the mitigation of fall risk, and improving patient outcomes.

## References

[1] Peterson M Greenwald BD. Balance problems after traumatic brain injury. Arch Phys Med Rehabil 2015; 96: 379–380. 25618196 10.1016/j.apmr.2013.06.012

[2] Rubenstein L Josephson K. The epidemiology of falls and syncope. Clin Geriatr Med 2002; 18: 141–158. 12180240 10.1016/s0749-0690(02)00002-2

[3] Gieger R Allen J O’Keefe J, et al Balance and mobility following stroke: effects of physical therapy interventions with and without biofeedback/forceplate training. Phys Ther 2001; 81: 995–1005. 11276182

[4] Allen NE Schwarzel AK Canning CG. Recurrent falls in Parkinson’s disease: a systematic review. Parkinsons Dis. Epub Mar 5 2013. 10.1155/2013/906274 PMC3606768 23533953

[5] Buchman AS Bennett DA. Loss of motor function in preclinical Alzheimer’s disease. Expert Rev Neurother 2011; 11: 665–676. 21539487 10.1586/ern.11.57 PMC3121966

[6] Stark SL Roe CM Grant EA, et al Preclinical Alzheimer disease and risk of falls. Neurology 2013; 81: 437–443. 23803314 10.1212/WNL.0b013e31829d8599 PMC3776538

[7] Rubenstein LZ. Falls in older people: epidemiology, risk factors and strategies for prevention. Age Ageing 2006; 35 (Suppl 2): ii37– ii41. 16926202 10.1093/ageing/afl084

[8] Burns ER Stevens JA Lee R. The direct costs of fatal and non-fatal falls among older adults—United States. J Safety Res 2016; 58: 99–103. 27620939 10.1016/j.jsr.2016.05.001 PMC6823838

[9] Nnodim JO Yung RL. Balance and its clinical assessment in older adults—a review. J Geriatr Med Gerontol 2015; 1: 1–19. 10.23937/2469-5858/1510003 PMC4773046 26942231

[10] Middleton A Fritz SL. Assessment of gait, balance, and mobility in older adults: considerations for clinicians. Curr Transl Geriatr Exp Gerontol Rep 2013; 2: 205–214.

[11] Schoene D Wu S Mikolaizak A, et al Discriminative ability and predictive validity of the timed up and go test in identifying older people who fall: systematic review and meta-analysis. J Am Geriatr Soc 2013; 61: 202–208. 23350947 10.1111/jgs.12106

[12] Kornetti DL Fritz SL Chiu YP, et al Rating scale analysis of the Berg balance scale. Arch Phys Med Rehabil 2004; 85: 1128–1135. 15241763 10.1016/j.apmr.2003.11.019

[13] Pardasaney P Latham N Jette A, et al Sensitivity to change and responsiveness of four balance measures for community-dwelling older adults. Phys Ther 2012; 92: 388–397. 22114200 10.2522/ptj.20100398 PMC3291380

[14] Miller Koop M Ozinga SJ Rosenfeldt AB, et al Quantifying turning behavior and gait in Parkinson’s disease using mobile technology. IBRO Reports 2018; 5: 10–16. 30135951 10.1016/j.ibror.2018.06.002 PMC6095098

[15] Salarian A Horak F Zampieri C, et al iTUG, a sensitive and reliable measure of mobility. IEEE Trans Neural Syst Rehabil Eng 2010; 18: 303–310. 20388604 10.1109/TNSRE.2010.2047606 PMC2922011

[16] Bernhard FP Sartor J Bettecken K, et al Wearables for gait and balance assessment in the neurological ward—study design and first results of a prospective cross-sectional feasibility study with 384 inpatients. BMC Neurol 2018; 18: 1–8. 30115021 10.1186/s12883-018-1111-7 PMC6094895

[17] Starling AJ Leong DF Bogle JM, et al Variability of the modified Balance Error Scoring System at baseline using objective and subjective balance measures. Concussion 2015; 1: CNC5, eCollection March 2016. 10.2217/cnc.15.5 PMC6114022 30202550

[18] Zhang C Truong M Talaber A, et al Quantitative comparison of K-D Balance and Sway BalanceTM system mobile applications. In: *American Academy of Neurology Sports Concussion Conference*, 2016.

[19] Downs S Marquez J Chiarelli P. Normative scores on the Berg Balance Scale decline after age 70 years in healthy community-dwelling people: a systematic review. J Physiother 2014; 60: 85–89. 24952835 10.1016/j.jphys.2014.01.002

[20] Nemmers T Miller J. Factors influencing balance in healthy community-dwelling women age 60 and older. J Geriatr Phys Ther 2008; 31: 93–100. 19856614 10.1519/00139143-200831030-00003

[21] de la Motte S Lisman P Gribbin T, et al . A systematic review of the association between physical fitness and musculoskeletal injury risk: part 3—flexibility, power, speed, balance, and agility. J Strength Cond Res 2019; 33(6): 1723–1735. 29239989 10.1519/JSC.0000000000002382

[22] Gribble PA Hertel J Plisky P. Using the star excursion balance test to assess dynamic postural-control deficits and outcomes in lower extremity injury: a literature and systematic review. J Athl Train 2012; 47: 339–357. 22892416 10.4085/1062-6050-47.3.08 PMC3392165

[23] Sandroff B Motl R Scudder M, et al Systematic, evidence-based review of exercise, physical activity, and physical fitness effects on cognition in persons with multiple sclerosis. Neuropsychol Rev 2016; 26: 271–294. 27447980 10.1007/s11065-016-9324-2

[24] Sosnoff J Moon Y Wajda D, et al Fall risk and incidence reduction in high risk individuals with multiple sclerosis: a pilot randomized control trial. Clin Rehabil 2015; 29: 952–960. 25540170 10.1177/0269215514564899

[25] Lieberman A Krishnamurthi N Dhall R, et al A simple question about falls to distinguish balance and gait difficulties in Parkinson’s disease. Int J Neurosci 2012; 122: 710–715. 22784291 10.3109/00207454.2012.711399

[26] Christofoletti G McNeely M Campbell M, et al Investigation of factors impacting mobility and gait in Parkinson disease. Hum Mov Sci 2016; 49: 308–314. 27551818 10.1016/j.humov.2016.08.007 PMC5026625

[27] Hsu Y Chung P Wang W, et al Gait and balance analysis for patients with Alzheimer’s disease using an inertial-sensor-based wearable instrument. IEEE J Biomed Heal Inform 2014; 18: 1822–1830. 10.1109/JBHI.2014.2325413 25375679

[28] Kenigsberg P Aquino J Berard A, et al Sensory functions and Alzheimer’s disease: a multi-disciplinary approach. Geriatr Psychol Neuropsychiatr Vieil 2015; 13: 243–258. 26395297 10.1684/pnv.2015.0553

[29] Hessam M Salehi R Yazdi M, et al Relationship between functional balance and walking ability in individuals with chronic stroke. J Phys Ther Sci 2018; 30: 993–996. 30154588 10.1589/jpts.30.993 PMC6110215

[30] Rand D. Mobility, balance and balance confidence—correlations with daily living of individuals with and without mild proprioception deficits post-stroke. NeuroRehabilitation 2018; 43: 219–226. 30175987 10.3233/NRE-172398

[31] Arkadir D Louis E. The balance and gait disorder of essential tremor: what does this mean for patients? Ther Adv Neurol Disord 2013; 6: 229–236. 23858326 10.1177/1756285612471415 PMC3707350

[32] Ludman H. Vertigo and imbalance. BMJ 2014; 348: 283. 10.1136/bmj.g283 24452766

[33] Ventura RE Balcer LJ Galetta SL. The neuro-ophthalmology of head trauma. Lancet Neurol 2014; 13: 1006–1016. 25231523 10.1016/S1474-4422(14)70111-5

[34] Chandrasekhar S. The assessment of balance and dizziness in the TBI patient. NeuroRehabilitation 2013; 32: 445–454. 23648599 10.3233/NRE-130867

[35] Muir SW Berg K Chesworth B, et al Balance impairment as a risk factor for falls in community-dwelling older adults who are high functioning: a prospective study. Phys Ther 2010; 90: 338–347. 20056721 10.2522/ptj.20090163

[36] Sibley KM Straus SE Inness EL, et al Clinical balance assessment: perceptions of commonly-used standardized measures and current practices among physiotherapists in Ontario, Canada. Implement Sci 2013; 8: 33. 23510277 10.1186/1748-5908-8-33 PMC3606836

[37] Downs S Marquez J Chiarelli P. The Berg Balance Scale has high intra- and inter-rater reliability but absolute reliability varies across the scale: a systematic review. J Physiother 2013; 59: 93–99. 23663794 10.1016/S1836-9553(13)70161-9

[38] Blum L Korner-Bitensky N. Usefulness of the Berg Balance Scale in stroke rehabilitation: a systematic review. Phys Ther 2008; 88: 559–566. 18292215 10.2522/ptj.20070205

[39] Pickenbrock H Diel A Zapf A. A comparison between the Static Balance Test and the Berg Balance Scale: validity, reliability, and comparative resource use. Clin Rehabil 2016; 30: 288–293. 25802425 10.1177/0269215515578297

[40] Kim S-G Kim M-K. The intra- and inter-rater reliabilities of the Short Form Berg Balance Scale in institutionalized elderly people. J Phys Ther Sci 2015; 27: 2733–2734. 26504281 10.1589/jpts.27.2733 PMC4616082

[41] Bower K McGinley J Miller K, et al Instrumented static and dynamic balance assessment after stroke using Wii Balance Boards: reliability and association with clinical tests. PLoS One 2014; 9: e115282. 25541939 10.1371/journal.pone.0115282 PMC4277284

[42] Howcroft J Kofman J Lemaire ED. Review of fall risk assessment in geriatric populations using inertial sensors. J Neuroeng Rehabil 2013; 10(1): 91. 23927446 10.1186/1743-0003-10-91 PMC3751184

[43] Zammit G Menz H Munteanu S. Reliability of the TekScan MatScan® system for the measurement of plantar forces and pressures during barefoot level walking in healthy adults. J Foot Ankle Res 2010; 3: 11. 20565812 10.1186/1757-1146-3-11 PMC2902454

[44] Brenton-Rule A Mattock J Carroll M, et al Reliability of the TekScan MatScan® system for the measurement of postural stability in older people with rheumatoid arthritis. J Foot Ankle Res 2012; 5: 21. 22889288 10.1186/1757-1146-5-21 PMC3431264

[45] Promsri A Haid T Federolf P. How does lower limb dominance influence postural control movements during single leg stance? Hum Mov Sci 2018; 58: 165–174. 29448161 10.1016/j.humov.2018.02.003

